# Supplementary material for: miR-197-3p Promotes Osteosarcoma Stemness and Chemoresistance by Inhibiting SPOPL
Source: J Clin Med. 2023 Feb 1;12(3):1177. doi: 10.3390/jcm12031177 (PMC9917813; doi:10.3390/jcm12031177)
Supplement: Supplementary file 1 [file jcm-12-01177-s001.zip › jcm-2155684-supplementary.pdf]

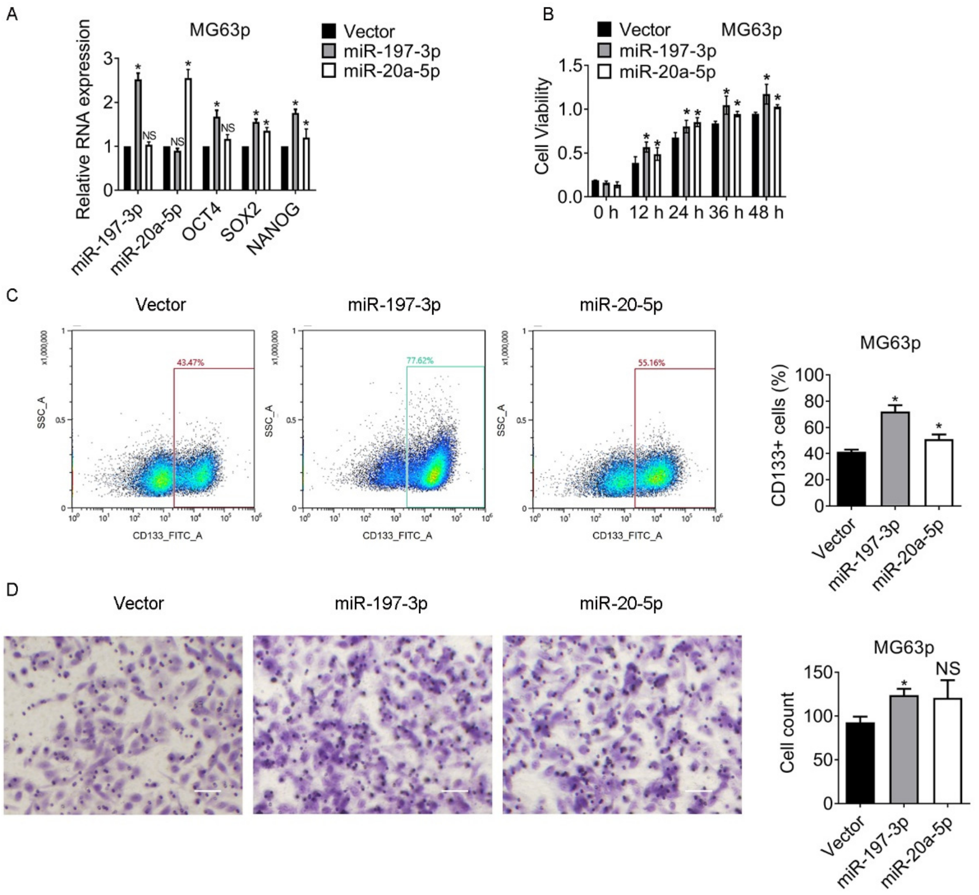

Supplementary Figure 1. miR-197-3p and miR-20a-5p promote chemoresistance and stemness of MTX-resistant MG63 cells. (**A**) The indicated miRNAs expression in the cell lines was assessed by qPCR assay. (**B**) The viability of the indicated cells was measured using MTT assays. (**C**) The quantity of CD133+ cells was then measured using flow cytometry. (**D**) The migration of the cells was assessed by transwell assay. Magnification, 200x. **P* < 0.05; NS, no significance. U2OSp, the parental U2OS; MG63p, the parental MG63. MG63p, the parental MG63.

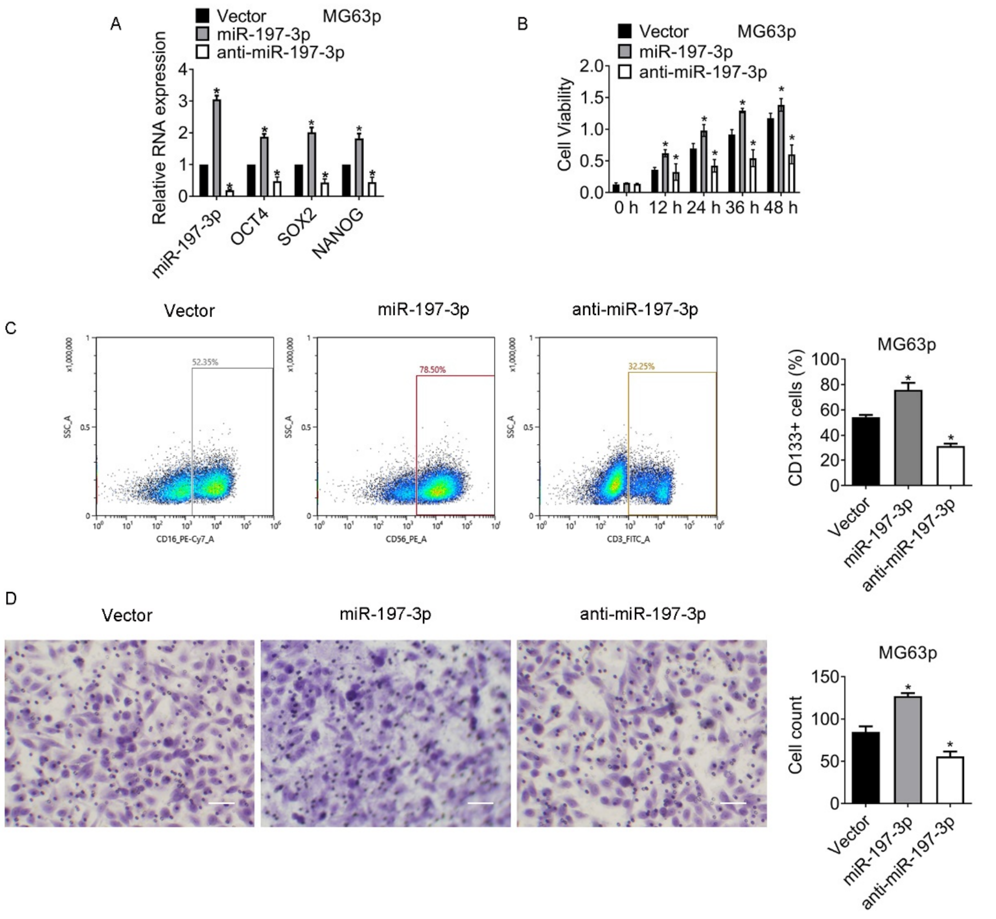

Supplementary Figure 2. miR-197-3p plays an essential role in maintaining chemoresistance and stemness of osteosarcoma cells MG63p. (**A**) The indicated genes expression was examined by qPCR and western blot. (**B**) The viability of the cells was assessed by MTT assay. (**C**) The quantity of CD133+ cells was measured by flow cytometry. (**D**) The migration of the cells was assessed by transwell assay. Magnification, 200x. *$P < 0.05$; NS, no significance. Results represented the mean ± S.D. of three independent experiments. MG63p, the parental MG63.

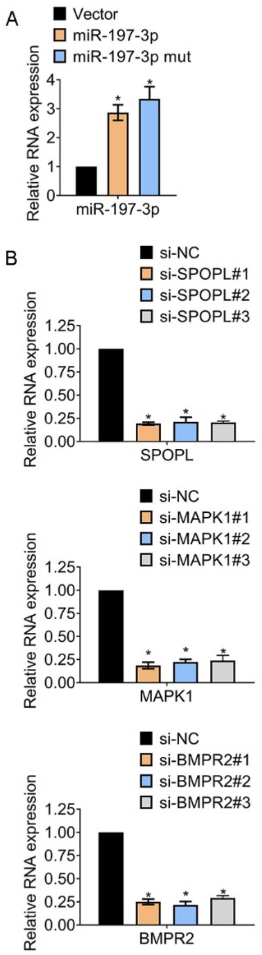

Supplementary Figure 3. The indicated miRNAs and mRNAs expression was determined by qPCR assay. (**A**) The overexpression of miR-197-3p and miR-197-3p mut was detected by qPCR assays. (**B**) The efficacy of silence RNAs was measured by qPCR assay. *$P < 0.05$; NS, no significance. Results represented the mean ± S.D. of three independent experiments.

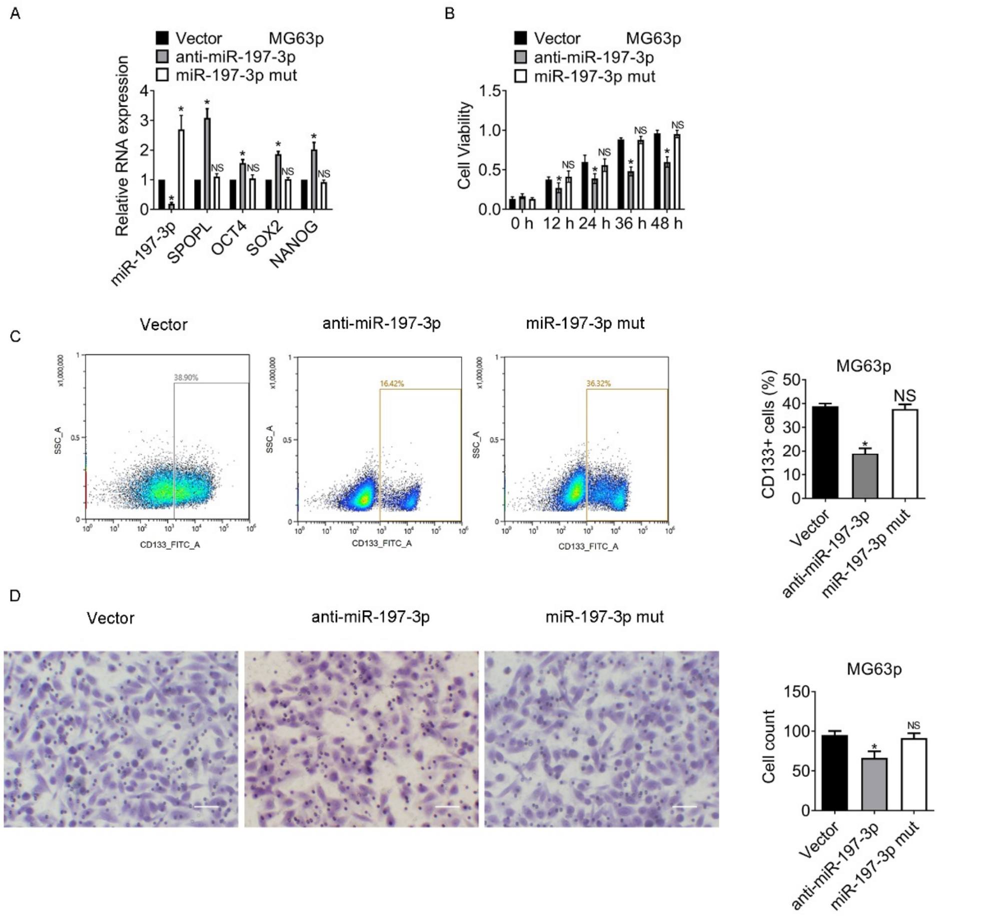

Supplementary Figure 4. miR-197-3p contributes to MG63 cells' chemoresistance and stemness by inhibiting SPOPL. (**A**) The indicated genes expression in the cells was detected by qRT-PCR and western blot. (**B**) The viability of the cells was assessed by MTT assay. (**C**) The representative images of CD133+ cells are shown. The CD133+ cells population was measured by flow cytometry. SSC-A indicates Side Scatter-Area. (**D**) The migration of the cells was assessed by transwell assay. Magnification, 200x. *$P < 0.05$; NS, no significance. Results represented the mean ± S.D. of three independent experiments. MG63p, the parental MG63.

Table S1. The association of miRNA expression and overall survival

miRNA	Overall survival (months)		*P* values
	Low expression	High expression	
miR-9-3p	85.83	48.87	0.0029*
miR-17-5p	86.63	41.17	8.1-e06*
miR-20a-5p	86.63	42.07	0.00012*
miR-124-3p	86.63	47.47	0.018*
miR-197-3p	54.23	22.93	0.000046*
miR-320d	77.47	61.5	0.44
miR-506-3p	81.6	49.27	0.13
miR-526b-3p	81.6	64.7	0.16

* Indicates *p* values < 0.05.

Table S2. The primers for detection list below.

miR-197-3p	Sense (5'-3')	TTCACCACCTTCTCCAC
	Reverse (5'-3')	GAACATGTCTGCGTATCTC
miR-20a-5p	Sense (5'-3')	GTGCTTATAGTGCAGGTA
	Reverse (5'-3')	GAACATGTCTGCGTATCTC
miR-17-5p	Sense (5'-3')	TGCTTACAGTGCAGGTAG
	Reverse (5'-3')	GAACATGTCTGCGTATCTC
SPOPL	Sense (5'-3')	TGGTCTTTTACCAGATGACAAGCT
	Reverse (5'-3')	AGACGACACTCAGGCACCTTCA
MAPK1	Sense (5'-3')	ACACCAACCTCTCGTACATCGG
	Reverse (5'-3')	TGGCAGTAGGTCTGGTGCTCAA
BMPR2	Sense (5'-3')	AGAGACCCAAGTTCCCAGAAGC
	Reverse (5'-3')	CCTTTCCTCAGCACACTGTGCA
OCT4	Sense (5'-3')	CCTGAAGCAGAAGAGGATCACC
	Reverse (5'-3')	AAAGCGGCAGATGGTCGTTTGG
SOX2	Sense (5'-3')	GCTACAGCATGATGCAGGACCA
	Reverse (5'-3')	TCTGCGAGCTGGTCATGGAGTT
NANOG	Sense (5'-3')	CTCCAACATCCTGAACCTCAGC
	Reverse (5'-3')	CGTCACACCATTGCTATTCTTCG
